# Preferences for HIV Testing Services and HIV Self-Testing Distribution Among Migrant Gay, Bisexual, and Other Men Who Have Sex With Men in Australia

**DOI:** 10.3389/fmed.2022.839479

**Published:** 2022-04-19

**Authors:** Ye Zhang, Virginia Wiseman, Tanya L. Applegate, Richard De Abreu Lourenco, Deborah J. Street, Kirsty Smith, Muhammad S. Jamil, Fern Terris-Prestholt, Christopher K. Fairley, Anna McNulty, Adam Hynes, Karl Johnson, Eric P. F. Chow, Benjamin R. Bavinton, Andrew Grulich, Mark Stoove, Martin Holt, John Kaldor, Rebecca Guy, Jason J. Ong

**Affiliations:** ^1^Kirby Institute, The University of New South Wales (UNSW) Sydney, Sydney, NSW, Australia; ^2^Department of Global Health and Development, London School of Hygiene and Tropical Medicine, London, United Kingdom; ^3^Centre for Health Economics Research and Evaluation, University of Technology Sydney, Sydney, NSW, Australia; ^4^Global Human immunodeficiency virus (HIV), Hepatitis and Sexually transmitted infections (STIs) Programmes, World Health Organization, Geneva, Switzerland; ^5^Central Clinical School, Faculty of Medicine, Nursing and Health Sciences, Monash University, Melbourne, VIC, Australia; ^6^Sydney Sexual Health Centre, Sydney, NSW, Australia; ^7^School of Population Health, The University of New South Wales (UNSW) Sydney, Sydney, NSW, Australia; ^8^Thorne Harbour Health, Melbourne, VIC, Australia; ^9^ACON, Sydney, NSW, Australia; ^10^Melbourne Sexual Health Centre, Alfred Health, Melbourne, VIC, Australia; ^11^Centre for Epidemiology and Biostatistics, Melbourne School of Population and Global Health, The University of Melbourne, Melbourne, VIC, Australia; ^12^Burnet Institute, Melbourne, VIC, Australia; ^13^Centre for Social Research in Health, The University of New South Wales (UNSW) Sydney, Sydney, NSW, Australia

**Keywords:** discrete choice experiment (DCE), migrants, men who have sex with men—MSM, HIV testing, HIV self-testing (HIVST), health preference research

## Abstract

**Background:**

In Australia, undiagnosed HIV rates are much higher among migrant gay, bisexual, or other men who have sex with men (GBMSM) than Australian-born GBMSM. HIV self-testing is a promising tool to overcome barriers to HIV testing and improve HIV testing uptake among migrant GBMSM. We compared the preferences for HIV testing services, including HIV self-testing, among migrant and Australian-born GBMSM.

**Methods:**

Preferences were assessed *via* two discrete choice experiments (DCEs). Participants were recruited between December 2017 and January 2018 using online and offline advertising and randomly assigned to complete one of two online DCE surveys. Migrant GBMSM were classified as being born in a country with a reciprocal healthcare agreement (RHCA) with Australia (providing free or subsided health care) or not. Latent class analysis and mixed logit models were used to explore heterogeneity in preferences.

**Findings:**

We recruited 1,606 GBMSM, including 583 migrant men of whom 419 (72%) were born in non-RHCA countries. Most participants preferred a free or cheap oral test with higher accuracy and a shorter window period to facilitate early detection of infections. Cost was more important for men born in non-RHCA countries than for men from RHCA countries or Australia. All groups preferred accessing kits through online distributers or off the shelf purchasing from pharmacies. Men born in RHCA countries least preferred accessing HIV self-testing kits from a medical clinic, while more than half of men from non-RHCA countries most preferred sourcing kits from a clinic. Sex-on-premises venues were the least preferred location to access test kits among all groups. In addition, two latent class analyses explored heterogeneity in preferences among men from non-RHCA countries and we found four latent classes for HIV testing services and two latent classes for HIVST distribution.

**Interpretation:**

Our findings emphasise the need for high-performing and low-cost HIV self-testing kits that are accessible from a variety of distribution points as a component of Australia's HIV response, especially for those who do not have access to free or subsidised health care in Australia.

## Introduction

Nearly one in 30 people in the world live in a country other than their place of birth (1). The focus on migrant health has been growing in recent years, with increasing recognition that the multi-faceted and heterogeneous nature of health risks, including those related to infectious disease prevention and control, can occur throughout the migration process (2). However, limited availability of quality data, and in some cases, access to publicly subsidised healthcare hamper efforts to address the health needs of migrants (3).

Over a quarter of people living in Australia (29.7%) were born overseas (4). In the early 2010s, migration-related HIV cases were predominantly diagnosed in people from sub-Saharan Africa (SSA), who acquired HIV before arriving in Australia (5). More recently, there has been an increase in the number of new HIV diagnoses in Australia among gay, bisexual or other men who have sex with men (GBMSM) from Asia (6). Migrants living in high-income countries are reported to have a lower self-perceived risk of HIV infection, language barriers, and concerns with confidentiality and privacy (7–10). Moreover, migrant GBMSM may face additional cultural and healthcare system barriers such as unfamiliarity with the local health system, distrust of health providers, and privacy concern, further hindering their access to HIV testing in conventional settings (11–13). Between 2014 and 2019, HIV diagnoses among Australian-born GBMSM declined by 44%, which likely occurred due to improved coverage of HIV testing and treatment, and implementation of PrEP across Australia (14). However, this success was offset by increased diagnoses among people born in other countries resulting in a relatively stable number of annual diagnoses overall (15). Surveillance data from 2018 suggest that migrant GBMSM in Australia are three times more likely to be undiagnosed for HIV and three times more likely to be diagnosed late than Australian-born GBMSM (14). Delays in testing lead to delayed treatment (16), and untreated HIV infections can disproportionately contribute to HIV transmissions (17–19). Together, this evidence underscores the urgent need to develop a better understanding of the needs of migrant GBMSM and improve access to earlier HIV testing, diagnosis, linkage to care, and bolster targeted prevention strategies.

Medicare, Australia's universal health care scheme, is available to all Australian citizens and permanent residents. Medicare also covers temporary migrants from ten countries in Europe and New Zealand through reciprocal healthcare agreements (RHCA) (20). With access to Medicare, migrants from RHCA countries have full access to free HIV testing. In comparison, migrants from non-RHCA countries can access free testing through public-funded programmes that are unevenly distributed across Australia or private insurance, which may require reimbursable upfront payments (21, 22). Previous studies found that migrants ineligible for subsidised healthcare through RHCA were more likely to be diagnosed with HIV later than those born in Australia or countries covered by the RHCA agreement (23, 24). In addition, studies on HIV and hepatitis B virus care in Australia have demonstrated that the issue of ineligibility for subsidised healthcare places additional psychological and financial pressure on migrants (25, 26).

HIV self-testing (HIVST) enables people to test for HIV conveniently and privately and is a promising tool to improve HIV testing uptake among migrant GBMSM (27, 28). Studies from several countries have confirmed that access to HIVST kits increases HIV testing uptake and frequency among GBMSM (29–32). More recently, HIVST kits have been successfully implemented in several countries to test underserved populations during the COVID-19 pandemic (33–35). To initiate or scale-up HIVST among GBMSM, various approaches to distribute HIVST kits have been evaluated globally, including through home delivery, pharmacies, vending machines, online purchasing, sexual or social networks, or social enterprise health campaigns—all of which have shown favourable outcomes (36–38). However, globally, little is known about the preferences of migrant GBMSM for accessing HIVST through these different channels.

Discrete choice experiments (DCEs) are a methodology to understand user preferences for goods and services that are not yet widely available in the market (39). Within a DCE, individuals are asked to state their preference between different goods or services on offer, with each of the goods or services described by their underlying characteristics or attributes (40). Data from DCEs can be used to identify the trade-offs that individuals are willing to make between the attributes describing a good or service (41). This method has been widely employed to quantitatively estimate user and provider preferences towards HIV testing services in various settings (42–46). In this paper, we compare the preferences for HIV testing services and HIVST kit distribution among GBMSM in Australia, assessing the impact on preferences of differences in country of birth and access to an RHCA for Medicare.

## Methods

### Study Population and Recruitment Procedures

This study used data from two DCEs (DCE-Test and DCE-Kits) administered through an online survey of GBMSM living in Australia (45). The recruitment procedures and details of the primary study are reported elsewhere (47). In brief, both surveys recruited GBMSM living in Australia, who were aged 18 years or over, and had not been diagnosed with HIV. Participants were recruited from December 2017 to January 2018 using online and offline advertising. The survey link was advertised in a dating application for GBMSM (Grindr) and social networking platforms, including the Facebook pages of two community-based organisations in Australia (ACON and Thorne Harbour Health, Australia's largest community-based HIV and LGBTI health organisations). For offline recruitment, GBMSM who attended the two largest public sexual health clinics in Sydney and Melbourne were invited by healthcare workers. In this study, migrants were defined as overseas-born people residing in Australia permanently or temporarily. Participants' sociodemographic characteristics and sexual histories were collected in the surveys, including their age, occupation, country of birth, duration of stay in Australia (if they were born outside Australia), condomless anal sex in the last 6 months, and HIV testing history. In this study, we focused our analyses primarily on migrant GBMSM and compared their preferences to men born in Australia. This study obtained ethical approval from the New South Wales South Eastern Sydney Local District Human Research Ethics Committee (17/147) and Alfred Health Human Research Ethics Committee (486/17).

### Design of the DCE and Selection of Attributes

After obtaining informed consent, participants were randomly assigned to complete one of two online DCE surveys (in a 2:1 ratio for DCE-Test and DCE-Kits). All surveys were in English. Attributes and levels for the DCEs were based on a review of the literature, qualitative interviews, and policy review (47). In the first DCE, participants were asked to choose between different aspects of HIV testing services to identify preferences for HIVST relative to other methods of HIV testing (DCE-Test). These attributes included the cost of the test, the length of the window period, the length of time to receive results, the accuracy of the HIV test, the type of sample used for testing, and who was responsible for collecting the sample. The second DCE examined preferences related to HIVST kit distribution (DCE-Kits). These attributes included the cost of the HIVST kit, where the kits were distributed, type of packaging, and type of instructions for use.

### Statistical Analysis

Participants' sociodemographic characteristics, sexual behaviours and HIV testing behaviours were characterised using descriptive statistics and compared between Australian-born and migrant GBMSM using Mann-Whitney *U*-tests for continuous variables and chi-squared tests for categorical variables. These analyses were conducted using Stata version 14 (StataCorp, College Station, Texas, USA).

Effects coding was used for all preference data. Mixed logit and latent class analysis models were used to estimate the relative utility of each attribute level (48). The mixed logit model assumed a continuous, normal distribution for all attributes, while the latent class analysis model assumed a discrete distribution based on latent constructs (49). We conducted two mixed logit models (one for HIV testing, one for HIVST distribution) that includes an interaction term for each attribute level to examine the differences in preferences for HIV testing services among Australian born men (Australian-born group), migrants from RHCA countries (RHCA country group) and migrants not from RHCA countries (non-RHCA country group). All attributes were set as random in the mixed logit model and treated as ordinal. In addition, the coefficient range of the levels for each attribute was used to calculate the relative weight of the attributes: the attribute with the largest range is likely to be the most important attribute in influencing testing behaviours (48). Latent class analysis models classify groups of responses that indicate homogenous preference patterns according to unobserved (latent) constructs; these groups can then be further characterised by using observable respondent characteristics to test the likelihood of respondents contributing to each class of response (49). We conducted two latent class analysis models to examine the heterogeneity of preferences for HIV testing services and HIVST distribution among the non-RHCA country group.

Several sociodemographic and sexual behaviour characteristics of participants recognised as key determinants for HIV testing in the published literature (23, 50, 51) were included in the latent class analysis models: young migrant (age ≤ 25-year-old); born in Southeast Asia; recent migrant (arriving Australia <5 years ago); ever engaged in condomless anal intercourse with casual partners in the last 6 months, and naive HIV tester (never tested for HIV). We used the participant's country of birth to determine if they were born in a country that has an RHCA with Australia, referred to as an RHCA country vs. a non-RHCA country (23, 24). The log-likelihood and Akaike Information Criteria (AIC) were used to evaluate the goodness-of-fit of the models. All mixed logit and latent class analysis models were estimated using NLOGIT statistical software (version 6, Econometric Software Inc, Plainview, NY, USA).

### Role of the Funding Source

The funder had no role in study design, data collection and analysis, or manuscript preparation.

## Results

Of the 1,606 men recruited between December 2017 and January 2018, 583 were migrants born overseas, with 420 and 163 participated in the DCE-Test survey and the DCE-kits survey, respectively. Table 1 presents the participants' sociodemographic characteristics, sexual behaviours, and HIV testing behaviours. Among the 419 (72%) migrant men born in non-RHCA countries, 127 (30%) were from Southeast Asia, 101 from other Asian countries (24%), 25 from Sub-Saharan Africa (6%) and 166 from other non-RHCA countries (40%). For migrants, the median time since arrival in Australia was seven years. Overall, migrant participants were younger and had higher education levels than those born in Australia. Compared to Australian-born men, a lower proportion of migrants had tested for HIV at a general practise and a higher proportion had tested at community services. In addition, 141 (24%) migrant men and 197 (19%) Australian-born men reported having delayed their HIV testing because no HIVST was available.

**Table 1:** Sociodemographic characteristics of all respondents completing DCETest (HIV testing method) and DCEKits (HIVST access), 2018 (*N* = 1,606) compared with Australia born GBMSM and migrant GBMSM.

	**DCE-Test survey**				**DCE-Kits survey**			
	**Total** ***N* = 1,168**	**Aust-GBM** ***N* = 748**	**Migrant group** ***N* = 420**	***P*-values**	**Total** ***N* = 438**	**Aust-GBM** ***N* = 275**	**Migrant group** ***N* = 163**	***P*-values**
**Sociodemographic characteristics**								
Mean age (SD)	36.1 (11.6)	36.5 (12.3)	35.3 (10.3)	<0.001^**^	35.9 (11.9)	37.3 (13.1)	33.4 (9.1)	<0.001^**^
Median age (IQR)	34 (27–43)	34 (27–45)	33 (27–41)		33 (27–44)	34 (26–47)	31 (27–39)	
Born in RHCA country			122 (29.0%)				42 (25.8%)	
Born in non-RHCA country								
Southeast Asia			95 (22.6%)				32 (19.6%)	
Other Asian country			69 (16.4%)				32 (19.6%)	
Sub-Saharan Africa			17 (4.0%)				8 (4.9%)	
Other non-RHCA countries			117 (13.1%)				49 (12.9%)	
**Median time since arrival in Australia**								
**Highest educational attainment**								
Up to Year 12	224 (19.2%)	180 (14.1%)	44 (10.5%)	<0.001^**^	86 (19.6%)	70 (25.5%)	16 (9.8%)	<0.001^**^
Trade certified, TAFE	222 (19.0%)	170 (22.7%)	52 (12.4%)	<0.001^**^	78 (17.8%)	63 (22.9%)	15 (9.2%)	<0.001^**^
University degree	722 (61.8%)	398 (53.2%)	324 (77.1%)	<0.001^**^	274 (62.6%)	143 (51.6%)	132 (81.0%)	<0.001^**^
**Employment status**								
Fulltime	699(59.9%)	454 (60.7%)	245 (58.3%)	0.43	276 (63.0%)	171 (62.2%)	105 (64.4%)	0.64
Part-Time	191 (16.4%)	110 (14.7%)	81 (19.3%)	0.05^*^	67 (15.3%)	39 (14.2%)	28 (17.2%)	0.40
Unemployed	91 (7.8%)	53 (7.1%)	38 (9.0%)	0.23	27 (6.2%)	21 (7.6%)	6 (3.7%)	0.10
Student	147 (12.6%)	95 (12.7%)	52 (12.4%)	0.87	60 (13.7%)	27 (9.8%)	33 (20.2%)	<0.002^**^
Pensioner	38 (3.3%)	34 (4.6%)	4 (1.0%)	<0.001^**^	10 (2.3%)	10 (3.6%)	0	<0.016^*^
**Sexual identity**								
Gay/homosexual	904 (77.4%)	576 (77.0%)	328(78.1%)	0.72	347 (79.2%)	218 (79.3%)	129 (79.1%)	0.97
Bisexual	212 (18.2%)	134 (17.9%)	78 (18.6%)	0.78	67 (15.3%)	41 (14.9%)	26 (16.0%)	0.77
Straight/heterosexual	18 (1.5%)	13 (1.7%)	5 (1.2%)	0.47	2 (0.5%)	1 (0.4%)	1 (0.6%)	0.99
Queer	22 (1.9%)	18 (2.4%)	4 (1.0%)	0.11	17 (3.9%)	10 (3.6%)	7 (4.3%)	0.75
Other	12 (1.0%)	7 (0.9%)	5 (1.2%)	0.76	5 (1.1%)	5 (1.8%)	0	0.16
**Sexual behaviours**								
Median number of anal sex partners in the last 6 months (IQR)	4 (2–9)	4 (1–9)	4 (2–8)		4 (1–8)	4 (1–8)	4 (2–8)	
Group sex in the last 6 months	478 (40.9%)	320 (42.8%)	158 (37.7%)	0.09	194 (44.3%)	120 (43.6%)	74 (45.4%)	0.72
Median number of casual anal sex partners in the last 6 months (IQR)	2 (2–2)	2 (2–2)	2 (2–2)		2 (1–2)	2 (2–2)	2 (2–2)	
Always used condoms with casual anal sex partner in the last 6 months (missing value)	329 (35.7%)	183(24.5%)	146 (34.8%)	<0.001^**^	124 (36.1%)	74 (26.9%)	50 (30.7%)	0.40
Used PreP for HIV in the last 6 months	253 (21.7%)	171 (22.9%)	82 (19.5%)	0.18	108 (24.7%)	71 (25.8%)	37 (22.7%)	0.46
**HIV testing behaviours**								
Ever tested for HIV	1,062 (90.9%)	670 (89.6%)	386 (91.9%)	0.19	394 (90.0%)	243 (88.4%)	149 (91.4%)	0.31
Never tested for HIV	106 (8.1%)	68 (10.4%)	34(8.1%)	0.19	44 (10%)	32 (11.6%)	14 (8.6%)	0.31
Used HIV self-test kit before	89 (8.4%)	40 (5.4%)	49 (11.7%)	<0.001^**^	27 (6.9%)	16 (5.8%)	11 (6.7%)	0.70
Put off HIV testing in the past year because no HIV self-test available	253 (23.9%)	148 (19.8%)	105 (25%)	0.04^*^	85 (21.7)	49 (17.8%)	36 (22.1%)	0.27
**Where participant normally attends for HIV test#**								
General practise	302 (28.4%)	221 (29.6%)	81 (19.3%)	<0.001^**^	110 (27.9%)	84 (30.6%)	26 (16.0%)	<0.001^**^
Community-based peer testing service	79 (7.3%)	34 (4.6%)	45(10.7%)	<0.001^**^	22 (5.6%)	10 (3.6%)	12 (7.4%)	0.08
Sexual health clinic	639 (60.2%)	405(54.1%)	234(55.7%)	0.60	243 (61.7%)	140 (50.9%)	103 (63.2%)	0.01^**^
Hospital	18 (1.7%)	3 (0.4%)	15 (3.6%)	<0.001^**^	6 (1.5%)	3 (1.1%)	3 (1.8%)	0.67
Other	18 (1.7%)	7 (0.9%)	11 (2.6%)	0.03^*^	11 (2.8%)	6 (2.2%)	5 (3.1%)	0.57
**Speed of receiving HIV results#**								
Same day	90 (8.5)	36 (4.8%)	54 (12.9%)	<0.001^**^	32 (8.1%)	19 (6.9%)	13 (8.0%)	0.68
2–3 days	424 (39.9%)	282 (37.7%)	142(33.8%)	0.18	147 (37.3%)	97 (35.3%)	50 (30.7%)	0.32
4–7 days	467 (44.0%)	303 (40.5%)	164 (39.0%)	0.62	182 (46.2%)	106 (38.6%)	76 (46.6%)	0.01^**^
More than a week	75 (7.1%)	49 (6.6%)	26 (6.2%)	0.39	31 (7.9%)	21 (7.6%)	10 (6.1%)	0.55

*SD, standard deviation; IQR, interquartile range;*

* *p < 0.05,*

** *p < 0.01;*

*DCETest, preferences of Australia-born and migrant GBMSM for HIV self-testing relative to other forms of HIV testing; DCEKits, preferences of Australia-born and migrant GBMSM for HIV self-testing distribution service; Aust-GBM, Australia-born GBMSM; RHCA country group, migrant GBMSM from RHCA eligible countries; non-RHCA country group, migrant GBMSM from RHCA-ineligible countries; PrEP, pre-exposure prophylaxis. #Only in men who have ever tested for HIV*.

### Comparison Between Groups: Preferences for HIV Testing Services (DCE-Test)

**Figure 1:**
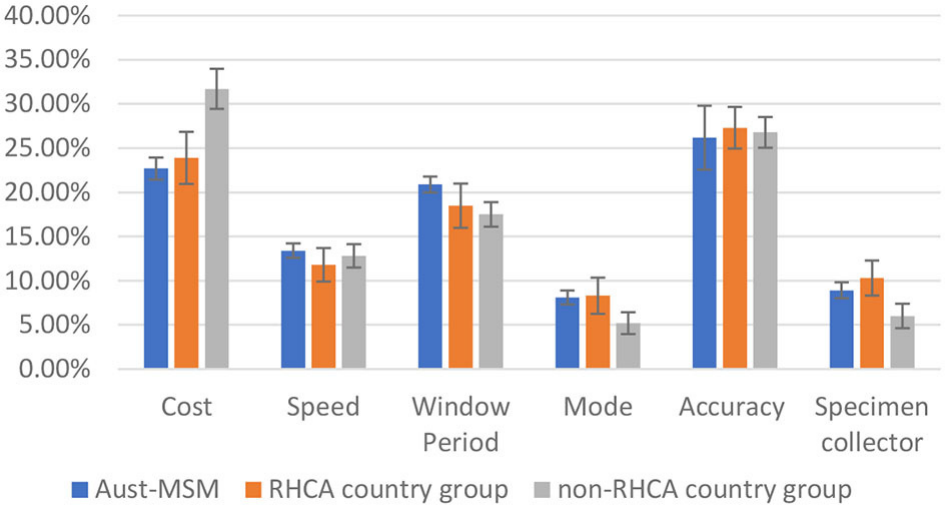
Relative importance of HIV testing attributes among Aust-MSM, RHCA country group, and non-RHCA country group.

Figure 1 shows the relative importance of each attribute among the Australian-born group, RHCA country group and non-RHCA country group according to the findings of the mixed logit models. The mixed logit model for the DCE-Test survey indicated that participants across the three groups generally preferred a free or low-cost oral test with higher accuracy and a shorter window period (Table 2, **Figure 3**). Notably, men from the non-RHCA country group expressed a significantly stronger preference for a free test kit compared to men in Australian-born and RHCA country groups (β = 0.60, *p* < 0.01). The finding was also confirmed in the importance of attributes across the three groups. In the Australian-born and RHCA country groups, the attribute with the highest relative importance was the accuracy of the test, followed by the cost of the test, window period, speed of receiving HIV results, person conducting the test, and the way a test kit was obtained. However, the cost of the test was the most important attribute for the non-RHCA country group.

**Table 2:** Preferences for HIV testing (DCETest) using the mixed logit model.

	**Aust-GBM**		**Interaction**	
			**RHCA country group**	**Non-RHCA country group**
	**Coeff. (SE)**	**SD (SE)**	**Coeff. (SE)**	**Coeff. (SE)**
**Cost (AUD)**				
Free (Reference)	1.37 (0.09)^***^	1.17 (0.22)^***^	0.22 (0.22)	0.60 (0.16)^***^
$20	0.27 (0.06)^***^	0.33 (0.09)^***^	0.16 (0.15)	0.04 (0.10)
$40	−0.40 (0.06)^***^	0.47 (0.12)^***^	−0.35 (0.17)^**^	−0.31 (0.11)^***^
$60	−1.24 (0.08)^***^	1.02 (0.07)^***^	−0.03 (0.20)	−0.33 (0.14)^**^
**Speed**				
1 min (Reference)	0.61 (0.05)^***^	0.55 (0.11)^***^	0.08 (0.12)	−0.01 (0.09)
20 min	0.49 (0.04)^***^	0.05 (0.07)	−0.22 (0.13)^**^	−0.09 (0.07)
1 day	−0.17 (0.04)^***^	0.21 (0.10)	−0.07 (0.11)	0.00 (0.08)
3 days	−0.93 (0.05)^***^	0.51 (0.05)^***^	0.21 (0.16)^*^	0.10 (0.08)
**Window period**				
4 weeks (Reference)	1.07 (0.05)^***^	0.98 (0.08)^***^	−0.11(0.14)	−0.22 (0.09)^**^
6 weeks	0.25 (0.03)^***^	0.03 (0.06)	0.04 (0.09)	0.00 (0.06)
3 months	−1.33 (0.06)^***^	0.98 (0.05)^***^	0.07 (0.14)	0.22 (0.09)^**^
**Mode**				
Venepuncture (Reference)	−0.58 (0.05)^***^	1.03 (0.07)^***^	0.05 (0.14)	0.25 (0.10)^**^
Oral	0.35 (0.05)^***^	0.68 (0.05)^***^	0.11 (0.12)	−0.10 (0.08)
Finger-prick	0.23 (0.04)^***^	0.36 (0.06)^***^	−0.16 (0.10)	−0.15 (0.07)^**^
**Accuracy**				
920 out of 1,000 (Reference)	−1.57 (0.07)^***^	1.20 (0.10)^***^	−0.19 (0.17)	0.01 (0.11)
950 out of 1,000	−0.49 (0.04)^***^	0.01 (0.06)	−0.00 (0.11)	−0.06 (0.07)
990 out of 1,000	0.62 (0.04)^***^	0.45 (0.06)^***^	0.12 (0.12)	0.06 (0.08)
999 out of 1,000	1.44 (0.06)^***^	1.11 (0.06)^***^	0.07 (0.16)	−0.01 (0.10)
**Specimen collected by**				
Healthcare worker (Reference)	−0.37 (0.06)^***^	0.97 (0.07)^***^	−0.13 (0.15)	0.09 (0.10)
Yourself	0.65 (0.05)^***^	0.83 (0.05)^***^	0.09 (0.14)	−0.26 (0.10)^**^
Peer	−0.28 (0.05)^***^	0.50 (0.06)^***^	0.04 (0.12)	0.17 (0.09)^*^

*Log-likelihood value −8761.3, AIC/N 0.944.*

* *p < 0.10,*

** *p < 0.05,*

*** *p < 0.01;*

*Coeff, coefficient; SE, standard error; AIC, Akaike information Criteria; Aust-GBM, Australia-born GBMSM; RHCA country group, migrant GBMSM from RHCA eligible countries; non-RHCA country group, migrant GBMSM from RHCA-ineligible countries*.

Furthermore, although respondents across the three groups all preferred HIV self-testing over testing by health workers or peers, those from the non-RHCA country group were significantly less likely to choose self-testing than men in the other two groups (β = −0.26, *p* < 0.05).

### Comparison Between Groups: Preferences for HIVST Distribution (DCE-Kits)

**Figure 2:**
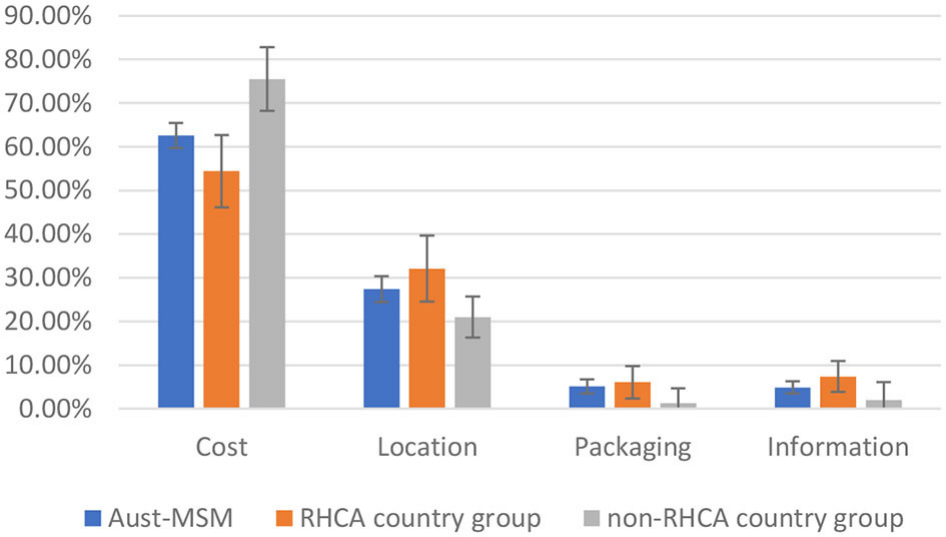
Relative importance of HIV-self testing distribution attributes among Aust-MSM, RHCA-eligible group, and RHCA-ineligible group.

**Figure 3:**
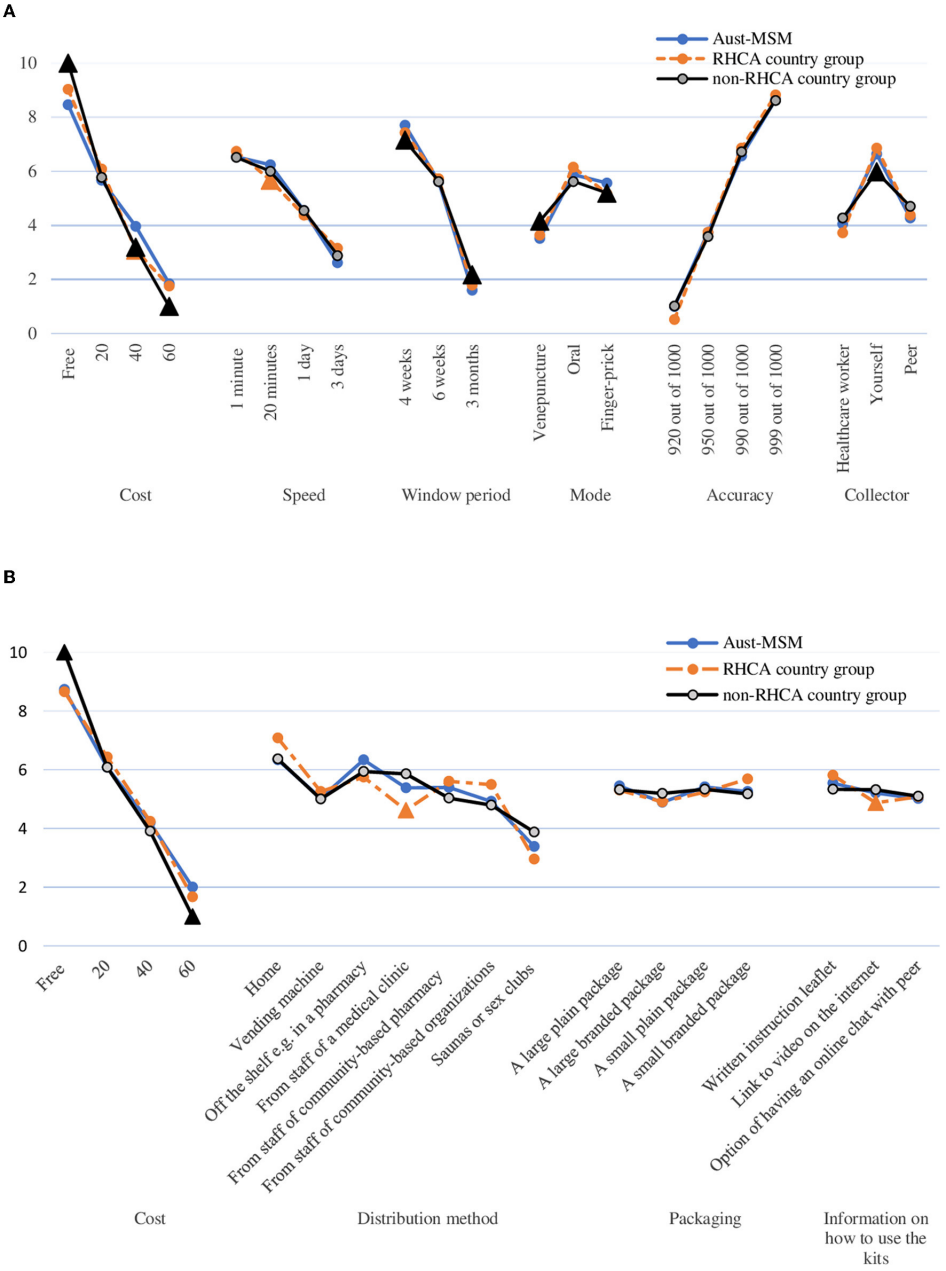
Scales estimated of HIV testing and HIV self-testing attributes among Aust-MSM, RHCA country group and non-RHCA country group. **(A)** Scaled estimates of HIV testing preferences, by country of birth. **(B)** Scaled estimates of HIV self-testing distribution preferences, by country of birth. Note: Triangle denotes estimate is statistically significantly different from the mean preference of Aust-MSM.

Table 3 show the mixed logit model results for the DCE-Kits survey, and Figure 2 shows the relative importance of each attribute. The most important attribute for all groups was the cost of the test, with a preference for low-cost self-testing kits. As in the results for the DCE-Test survey, men from the non-RHCA country group expressed a significantly stronger preference for a free self-test kit than those in Australian-born and RHCA country groups (β = 0.99, *p* < 0.01). The location where HIVST kits could be accessed was the second most important attribute. All groups preferred accessing kits through online distributors or off the shelf purchasing from pharmacies, less preferred getting kits from the staff of a community-based organisation, and getting kits from sex-on-premises-venues was least preferred. Men from the RHCA country group least preferred kits from medical clinics compared with men from non-RHCA country and Australian-born groups (β = −0.59, *p* < 0.05). On the contrary, the non-RHCA country group were more likely to access kits at a medical clinic, although the difference between this group and the Australian-born group was not significant. We also found differences between the groups in their preferences for usage instructions. For participants from the RHCA country group, accessing usage instruction through watching a video online was less preferred (β = −0.26, *p* < 0.05; Figure 3).

**Table 3:** Preferences for HIV self-testing distribution (DCEKits) using the mixed logit model.

	**Aust-GBM**		**Interaction**	
			**RHCA country group**	**Non-RHCA country group**
	**Coeff. (SE)**	**SD (SE)**	**Coeff. (SE)**	**Coeff. (SE)**
**Cost (AUD)**
Free (Reference)^#^	2.71 (0.16)^***^	1.84 (0.25)^***^	−0.06 (0.34)	0.99 (0.27)^***^
$20	0.65 (0.07)^***^	0.07 (0.17)	0.27 (0.18)	0.00 (0.12)
$40	−0.83 (0.08)^***^	0.68 (0.09)^***^	0.04 (0.21)	−0.22 (0.14)
$60	−2.54 (0.15)^***^	1.71 (0.12)^***^	−0.25(0.32)	−0.77(0.24)^***^
**Distribution method**
Order online with kits mailed to home (Reference)^#^	0.84 (0.17)^***^	2.4 (0.59)^***^	0.58 (0.53)	0.03 (0.32)
Kits available from a public vending machine	−0.17 (0.11)	0.95 (0.12)^***^	0.17 (0.30)	−0.03 (0.21)
Kits available off the shelf e.g., in a pharmacy	0.84 (0.11)^***^	0.68 (0.13)^***^	−0.45 (0.29)	−0.31 (0.20)
Kits available from staff of a medical clinic	0.10 (0.10)	1.02 (0.13)^***^	−0.59 (0.30)^**^	0.37 (0.21)^*^
Kits available from staff of a community-based pharmacy	0.11 (0.10)	0.62 (0.13)^***^	0.17 (0.30)	−0.28 (0.21)
Kits available from staff of a community-based organisation	−0.26 (0.11)^**^	0.73 (0.11)^***^	0.45 (0.29)	−0.10 (0.21)
Kits available from staff of “saunas or sex clubs”	−1.46(0.16)^***^	1.56 (0.16)^***^	−0.33 (0.40)	0.38 (0.27)
**Packaging**
A large plain package (Reference)^#^	0.15 (0.10)^*^	0.42 (0.19)^**^	−0.11(0.24)	−0.10 (0.19)
A large branded package	−0.28 (0.07)^***^	0.31 (0.09)^***^	0.01 (0.18)	0.23 (0.13)
A small plain package	0.13 (0.07)^**^	0.14 (0.21)	−0.14 (0.18)	−0.07 (0.13)
A small branded package	−0.00 (0.07)	0.24 (0.13)^*^	0.34 (0.18)	−0.06 (0.13)
**Information on how to use the kits**
Written instruction leaflet (Reference)^#^	0.22 (0.05)^***^	0.43 (0.18)^***^	0.22 (0.15)	−0.15 (0.10)
Link to video on the internet	−0.04 (0.05)	0.21 (0.09)^***^	−0.26 (0.16)^**^	0.09 (0.10)
Option of having an online chat with peer	−0.18 (0.06)^***^	0.37 (0.06)^***^	0.04 (0.15)	0.06 (0.11)

*Log-likelihood value −2,858.10, AIC/N 0.832.*

* *p < 0.10,*

** *p < 0.05,*

*** *p < 0.01;*

# *The coefficient for the reference group is calculated as the negative sum of the other coefficients.*

*Coeff, coefficient; SE, standard error; AIC, Akaike information Criteria; Aust-GBM, Australia-born GBMSM; RHCA country group, migrant GBMSM from RHCA eligible countries; non-RHCA country group, migrant GBMSM from RHCA-ineligible countries*.

### Non-RHCA Group: Latent Class Analyses

**Figure 4:**
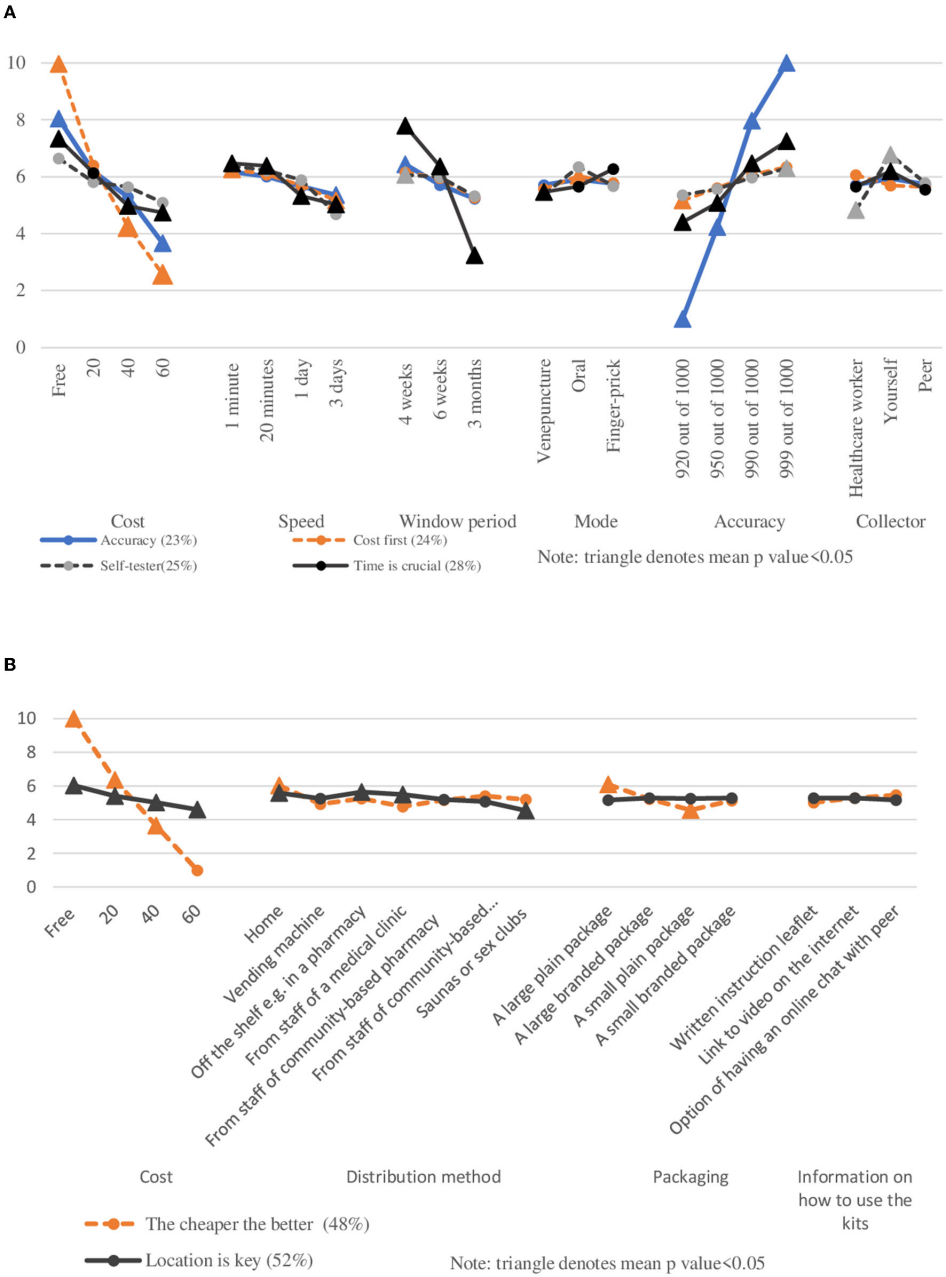
**(A)** Scaled estimates of HIV testing preferences among non-RHCA country group, by classes. **(B)** Scaled estimates of HIV self-testing distribution preferences among non-RHCA country group, by classes. Note: Triangle denotes mean *p* < 0.05

Given the small number of respondents born in RHCA countries, latent class analysis models were only created for respondents from the non-RHCA country group to further understand the heterogeneous preferences for HIV testing services and HIVST distribution in this group. The goodness-of-fit for the latent class analysis models are presented and compared in Supplementary Figures 1, 2. In the DCE-Test survey, 23% of participants belonged to Class 1 (“accuracy-oriented”). This group preferred free, faster, and more accurate tests with a shorter window period. Men in the second class (“cost first,” 24%) were most influenced by the cost of the test and tended to be aged above 25 years or were born in Southeast Asia. The second class preferred a more accurate oral test with a shorter window period. Men in Class 3 (“self-tester,” 25%) were strongly influenced by who collected the specimen. They preferred testing themselves and were more likely to be aged above 25 years. Men in Class 4 (“timing is crucial,” 28%) were most sensitive towards the test window period. They preferred a free, faster, and more accurate test with a shorter window period (Table 4, Figure 4).

**Table 4:** Latent class analysis of HIV testing preferences of Australian migrant men who have sex with men born in non-RHCA countries (*N* = 298).

	**DCETests**			
	**Accuracy-Oriented** **(23%) Coeff. (SE)**	**Cost first** **(24%) Coeff. (SE)**	**Self-Tester** **(25%) Coeff. (SE)**	**Timing is crucial** **(28%) Coeff. (SE)**
**Cost (AUD)**				
Free (Reference)^#^	1.73 (0.30)^***^	3.21 (0.42)^***^	0.65 (0.15)^***^	1.18 (0.21)^***^
$20	0.30 (0.19)	0.46 (0.24)	0.01 (0.14)	0.26 (0.16)
$40	−0.39 (0.22)	−1.19 (0.20)^***^	−0.12 (0.14)	−0.63 (0.16)^***^
$60	−1.64 (0.29)^***^	−2.48 (0.22)^***^	−0.54 (0.16)^***^	−0.81 (0.18)^***^
**Speed**				
1 min (Reference)^#^	0.29 (0.18)	0.35 (0.11)^***^	0.45 (0.10)^***^	0.52 (0.13)^***^
20 min	0.16 (0.15)	0.22 (0.15)	0.33 (0.09)^***^	0.45 (0.13)^***^
1 day	−0.11 (0.17)	−0.08 (0.11)	0.07 (0.09)	−0.37 (0.13)^***^
3 days	−0.34 (0.17)^**^	−0.49 (0.11)^***^	−0.85 (0.11)^***^	−0.60 (0.12)^***^
**Window period**				
4 weeks (Reference)^#^	0.49 (0.13)^***^	0.28 (0.10)^***^	0.21 (0.09)^**^	1.53 (0.14)^***^
6 weeks	−0.06 (0.15)	0.13 (0.09)	0.15 (0.08)	0.43 (0.10)^***^
3 months	−0.43 (0.12)^***^	−0.41 (0.10)^***^	−0.36 (0.11)^***^	−1.96 (0.16)^***^
**Mode**				
Venepuncture (Reference)^#^	−0.06 (0.15)	−0.17 (0.12)	−0.33 (0.11)^***^	−0.26 (0.13)^**^
Oral	0.08 (0.20)	0.18 (0.09)^**^	0.42 (0.09)^***^	−0.11 (0.11)
Finger-Prick	−0.02 (0.21)	−0.01 (0.10)	−0.09 (0.10)	0.37 (0.12)
**Accuracy**				
920 out of 1,000 (Reference)^#^	−3.69 (0.30)^***^	−0.48 (0.10)^***^	−0.34 (0.10)^***^	1.07 (0.13)^***^
950 out of 1,000	−1.21 (0.19)^***^	−0.15 (0.12)	−0.18 (0.10)	−0.56 (0.12)^***^
990 out of 1,000	1.67 (0.20)^***^	0.21 (0.11)	0.14 (0.10)	0.52 (0.14)^***^
999 out of 1,000	3.23 (0.27)^***^	0.42 (0.12)^***^	0.38 (0.10)^***^	1.11 (0.11)^***^
**Specimen collected by**				
Healthcare worker (Reference)^#^	−0.06 (0.17)	0.20 (0.13)	−0.74 (0.11)^***^	−0.11 (0.12)
Yourself	0.11 (0.13)	−0.08 (0.10)	0.75 (0.11)^***^	0.30 (0.11)^***^
Peer	−0.05 (0.14)	−0.12 (0.10)	−0.01 (0.09)	−0.19 (0.12)
**Theta in class probability model**				
Young	−0.36 (0.25)	−1.18 (0.48)^**^	−0.57 (0.28)^**^	Reference
SEA	0.50 (0.39)	1.05 (0.49)^**^	0.06 (0.40)	Reference
SSA	−0.68 (0.60)	−1.43 (0.86)	−0.33 (0.55)	Reference
Never tested	0.53 (0.42)	0.26 (0.58)	0.32 (0.45)	Reference
Condomless	−0.32 (0.23)	−0.17 (0.25)	0.01 (0.24)	Reference

*Log-likelihood value −2,220.61, AIC/N 0.964.*

* *p < 0.05,*

*** *p < 0.01;*

# *The coefficient for the reference group is calculated as the negative sum of the other coefficients.*

*Coeff, coefficient; SE, standard error; AIC, Akaike information Criteria; non-RHCA country group, migrant GBMSM from RHCA-ineligible countries; PrEP, pre-exposure prophylaxis; SEA, South-East Asia; SSA, Sub Saharan Africa*.

In the DCE-Kits survey, nearly half of the respondents belonged to Class 1 (“the cheaper the better,” 48%) and were most influenced by the cost of the test. They had no significant preference for where to access the test but least preferred a test kit with a small plain package. Men who had never been tested were more likely to belong to this class. The second class (“location is key,” 52%) preferred kits purchased off the shelf in a pharmacy, followed by online distribution and asking for a kit from a clinic's staff. They least preferred accessing kits from sex-on-premises venues (Table 5, Figure 4).

**Table 5:** Latent class analysis of HIVST distribution preferences of Australian migrant men who have sex with men born in non-RHCA countries (*N* = 121).

	**DCEKits**	
	**The cheaper** **the better (48%)** **Coeff. (SE)**	**Location is** **key (52%) Coeff.** **(SE)**
**Cost (AUD)**		
Free (reference)^#^	5.74 (0.74)^***^	0.92 (0.11)^***^
$20	1.33 (0.33)^***^	0.18 (0.08)^**^
$40	−1.94 (0.30)^***^	−0.30 (0.08)^***^
$60	−5.14 (0.66)	−0.80 (0.10)^***^
**Access location**		
Order online with kits mailed to home (Reference)^#^	0.95 (0.47)^**^	0.4 (0.11)^***^
Kits available from a public vending machine	−0.40 (0.50)	−0.01 (0.13)
Kits available from the shelf e.g., in a pharmacy	−0.00 (0.51)	0.47 (0.13)^***^
Kits available from the staff of a medical clinic	−0.56 (0.56)	0.29 (0.12)^**^
Kits available from the staff of community-based pharmacy	−0.10 (0.47)	−0.07 (0.15)
Kits available from the staff of community-based organisations	0.17 (0.46)	−0.21 (0.14)
Kits available from the staff of “saunas or sex clubs”	−0.06 (0.48)	−0.87 (0.17)^***^
**Packaging**		
A large plain package (Reference)^#^	1.00 (0.40)^***^	−0.09 (0.08)
A large branded package	−0.05 (0.45)	0.05 (0.08)
A small plain package	−0.82 (0.39)^**^	0.01 (0.08)
A small branded package	−0.13 (0.28)	0.03 (0.08)
**Information on how to use the kits**		
Written instruction leaflet (Reference)^#^	−0.28 (0.24)	0.05 (0.06)
Link to video on the internet	0.02 (0.25)	0.05 (0.07)
Option of having an online chat with a peer	0.26 (0.24)	−0.10 (0.07)
**Theta in class probability model**		
Never tested	1.08 (0.65)^*^	Reference
Young	−0.20 (0.31)	Reference
SEA	0.45 (0.33)	Reference
SSA	−0.12 (0.65)	Reference
Condomless	−0.25 (0.32)	Reference

*Log-likelihood value −715.44; AIC/N 0.774.*

** *p < 0.05,*

*** *p < 0.01;*

# *The coefficient for the reference group is calculated as the negative sum of the other coefficients.*

*Coeff, coefficient; SE, standard error; AIC, Akaike information Criterianon; RHCA country group, migrant GBMSM from RHCA-ineligible countries; PrEP, pre-exposure prophylaxis; SEA, South-East Asia; SSA, Sub Saharan Africa*.

## Discussion

In light of the increasing number of HIV diagnoses among migrant GBMSM in Australia, expanding HIV testing coverage among this group is critical to prevent onward transmission in undiagnosed men. Previous research emphasises the need to identify testing strategies that effectively target migrants who may face various obstacles to accessing HIV testing (52). Our study extends the limited literature on HIV testing for migrant populations by identifying heterogeneous preferences towards HIV testing services. To our knowledge, this is the first study that informs optimisation of access and uptake of HIV self-testing among migrants, especially those who cannot easily access Medicare-subsidised health services.

Unlike Australian-born GBMSM and men from RHCA countries, the cost of an HIV test was the most important attribute for GBMSM from non-RHCA countries. This finding is consistent with previous studies that find that promoting free or reduced-cost HIV services among undertested people who have constrained access to healthcare improves their engagement in the HIV cascade (23, 53–56). In Australia, free HIV testing services are available through state government-funded sexual health centres and community-based health services, regardless of their Medicare status (21, 22). Raising awareness among migrants of services that offer free HIV care in Australia may help improve testing uptake. But, it is worth noting that free or low-cost services are not uniformly available across the country and tend to be concentrated in capital cities and regional centres.

Furthermore, given the preferences of all GBMSM, an ideal HIV testing kit would be a saliva-based test that has high accuracy, a short window period and allows them to test by themselves. However, albeit with a general preference for oral-fluid tests, currently there is no oral-fluid rapid test for self-testing in Australia that is regulatory approved. In addition, the oral self-tests are likely to be less sensitive than blood-based self-tests and all self-tests for HIV, regardless of whether it is saliva or blood-based test, have reduced sensitivity during the window period (57, 58). To date, the Australian Therapeutic Goods Administration (TGA) has approved only one HIVST that uses a blood sample (59). The availability of blood-based HIVST may appeal to those who value accuracy the most but may not be the kit those who prefer an easy-to-use and painless test want to purchase. Our study showed that oral testing is the preferred option for migrants from non-RHCA countries, especially older men born in South East Asia countries. Consequently, to reach this group of migrants, it is important to have different types of test kits available in the market to give them more choice.

Consistent with previous Australian research, our findings suggest that HIVST is acceptable to migrant men (60). In addition, compared to Australian GBMSM, a higher proportion of migrant men had previously used HIVST, and a higher percentage (about a quarter of men) reported that they had delayed HIV testing because HIVST was not available. The Atomo HIV self-test kit was approved for marketing in Australia at the end of 2018. The initial regulation restricted the sale of HIVST kits to online purchasing or through approved health organisations (59). Our findings from latent class models show that over half of non-RHCA migrants most preferred to get the HIVST kits through pharmacies, which could be ideal for those who do not want to make a medical appointment or wait for long periods before testing. Our results support the recent decision of Australian TGA to allow HIVST to be sold in pharmacies at the end of 2021. Further research is needed to fully understand the impact of changes in the accessibility of HIVST on its uptake.

In addition, migrants from the non-RHCA country group also showed a stronger preference for accessing the kits in medical clinics. In our study, nearly two-thirds of the men from the non-RHCA country group were born in Southeast Asia and Sub-Saharan Africa. Studies have shown that migrants from Sub-Saharan Africa and Asia often prefer some level of assistance to perform HIVST when they conduct their first or second test, as support from healthcare workers may ensure the accuracy of results (61–63). The majority of men in our study had not previously used an HIVST kit, which may explain a preference for assisted HIVST to ensure correct use, at least for the first time. Further studies are needed to understand the factors affecting migrant GBMSM from Asia and Africa to identify and respond to their specific needs and better tailor HIVST interventions to this group.

The strength of this study is its exploration of HIV testing preferences among migrant GBMSM compared with Australian-born GBMSM. Until this study, the availability of preference data from this underserved population was limited, mainly due to the small sample size of previous DCE studies and a lack of targeted recruitment of migrant GBMSM (46). Another strength is that we used the established methodology of DCE to quantitatively measure preference heterogeneity (64). There are a few limitations that should be noted. First, all surveys were presented in English, which might affect the generalizability of the findings to non-English speaking migrants. Language barriers could potentially impede migrants' access to healthcare in facilities (65). Our findings that migrants from the non-RHCA group preferred accessing HIVST in a clinic may not apply to migrants who do not speak English. Second, we did not assess men's residency status in Australia but used the eligibility for RHCA as a proxy for access to subsidised healthcare in Australia, similar to previous studies (23). Some men from the non-RHCA country group may have access to subsided healthcare after receiving permanent residency in Australia. If this is so, our result may underestimate the role of Medicare in determining the preferences of migrant men towards the cost of HIV testing services. Conversely, it is also possible that men from the RHCA eligible group, who are young and healthy, may not be aware of the scheme and therefore not register for temporary access to Medicare. Future research on migrants could include visa status and Medicare status to better understand differences among this population group.

In summary, our study indicates that low-cost and high performing self-testing kits might improve HIV testing among migrant GBMSM in Australia, especially those from non-RHCA countries. Our study highlights differences in HIV self-testing kit distribution preferences that can inform future HIVST implementation projects targeting migrants. However, until now, no HIVST kit distribution strategies have thus far been tailor-designed to reach migrant GBMSM in Australia. Providing targeted testing services and supports for migrant men is essential to get newly arrived men tested shortly after arrival to prevent onward transmission in this population. These findings emphasise the importance of expanding the availability and ease of access to multiple types of HIVST kits in Australia to optimise the uptake of HIVST among migrant men.

## Data Availability Statement

The original contributions presented in the study are included in the article/Supplementary Material, further inquiries can be directed to the corresponding author.

## Ethics Statement

This study obtained ethical approval from the New South Wales South Eastern Sydney Local District Human Research Ethics Committee (17/147) and Alfred Health Human Research Ethics Committee (486/17). The patients/participants provided their written informed consent to participate in this study.

## Author Contributions

The study was designed by YZ, JO, RL, DS, KS, MJ, AH, KJ, BB, MH, JK, and RG. YZ, JO, VW, RG, and FT-P were involved in data analysis and interpretation. YZ and JO contributed to the writing of the manuscript. All authors contributed to the interpretation results, provided advice of the draft, and approved the final draft of submission.

## Funding

This study was funded by the Australian National Health and Medical Research Council (Grant Nos. APP1104781 and 568971). EC and JO are supported by an Australian National Health and Medical Research Council (NHMRC) Emerging Leadership Investigator Grant (GNT1172873 and GNT1193955, respectively). CF was supported by an NHMRC Leadership Investigator Grant (GNT1172900).

## Author Disclaimer

The contents in this article are those of the authors alone and do not necessarily reflect the view of the World Health Organisation.

## Conflict of Interest

AH was employed by Thorne Harbour Health. KJ was employed by ACON. The remaining authors declare that the research was conducted in the absence of any commercial or financial relationships that could be construed as a potential conflict of interest.

## Publisher's Note

All claims expressed in this article are solely those of the authors and do not necessarily represent those of their affiliated organizations, or those of the publisher, the editors and the reviewers. Any product that may be evaluated in this article, or claim that may be made by its manufacturer, is not guaranteed or endorsed by the publisher.
