# Supplementary material for: Preferences for HIV Testing Services and HIV Self-Testing Distribution Among Migrant Gay, Bisexual, and Other Men Who Have Sex With Men in Australia
Source: Front Med (Lausanne). 2022 Apr 19;9:839479. doi: 10.3389/fmed.2022.839479 (PMC9063480; doi:10.3389/fmed.2022.839479)
Supplement: Supplementary file 1 [file Data_Sheet_1.docx]

**Supplementary Figure S1. Model fit using Akaike Information Criteria according to the number of classes for DCETest (HIV testing preferences)**

We chose to present the four-class model in our study as the models with five classes or above did not provide any further useful patterns of preferences that would be useful to a policy-maker and showed extreme standard error.

**Supplementary Figure S2. Model fit using Akaike Information Criteria according to the number of classes for DCEKits (HIVST access preferences)**

We chose to present the two-class model in our study as the models with three classes or above did not provide any further useful patterns of preferences that would be useful to a policy-maker and showed extreme standard error.
